# Integrative analysis of the expression profiles of whole coding and non-coding RNA transcriptomes and construction of the competing endogenous RNA networks for chronic obstructive pulmonary disease

**DOI:** 10.3389/fgene.2023.1050783

**Published:** 2023-01-30

**Authors:** Xueyan Feng, Hui Dong, Beibei Li, Liang Yu, Jinyuan Zhu, Caili Lou, Jin Zhang

**Affiliations:** ^1^ Clinical medical school, Ningxia Medical University, Yinchuan, China; ^2^ Institute of Medical Sciences, General Hospital of Ningxia Medical University, Yinchuan, China; ^3^ Department of Thoracic Surgery, General Hospital of Ningxia Medical University, Yinchuan, China; ^4^ Department of Critical Care Medicine, General Hospital of Ningxia Medical University, Yinchuan, China; ^5^ Department of Respiratory and Critical Care Medicine, General Hospital of Ningxia Medical University, Yinchuan, China

**Keywords:** chronic obstructive pulmonary disease (COPD), circular RNA (circRNA), long non-coding RNA (IncRNA), MicroRNA (miRNA), messenger RNA (mRNA), competing endogenous RNAs (ceRNA) network

## Abstract

The pathogenesis of Chronic Obstructive Pulmonary Disease (COPD) is implicated in airway inflammation, oxidative stress, protease/anti-protease and emphysema. Abnormally expressed non-coding RNAs (ncRNAs) play a vital role in regulation of COPD occurrence and progression. The regulatory mechanisms of the circRNA/lncRNA-miRNA-mRNA (competing endogenous RNA, ceRNA) networks might facilitate our cognition of RNA interactions in COPD. This study aimed to identified novel RNA transcripts and constructed the potential ceRNA networks of COPD patients. Total transcriptome sequencing of the tissues from patients with COPD (COPD) (*n* = 7) and non-COPD control subjects (Normal) (*n* = 6) was performed, and the expression profiles of differentially expressed genes (DEGs), including mRNAs, lncRNAs, circRNAs, and miRNAs, were analyzed. The ceRNA network was established based on the miRcode and miRanda databases. Kyoto Encyclopedia of Genes and Genomes (KEGG), Gene Ontology (GO), Gene Set Enrichment Analysis (GSEA), and Gene set variation analysis (GSVA) were implemented for functional enrichment analysis of DEGs. Finally, CIBERSORTx was extracted to analyze the relevance between hub genes and various immune cells.The Starbase and JASPAR databases were used to construct hub-RNA binding proteins (RBPs) and lncRNA-transcription factor (TF) interaction networks. A total of 1,796 mRNAs, 2,207 lncRNAs, and 11 miRNAs showed differentially expression between the lung tissue samples from the normal and COPD groups. Based on these DEGs, lncRNA/circRNA-miRNA-mRNA ceRNA networks were constructed respectively. In addition, ten hub genes were identified. Among them, RPS11, RPL32, RPL5, and RPL27A were associated with the proliferation, differentiation, and apoptosis of the lung tissue. The biological function revealed that TNF–α via NF–kB and IL6/JAK/STAT3 signaling pathways were involved in COPD. Our research constructed the lncRNA/circRNA-miRNA-mRNA ceRNA networks, filtrated ten hub genes may regulate the TNF-α/NF-κB, IL6/JAK/STAT3 signally pathways, which indirectly elucidated the post-transcriptional regulation mechanism of COPD and lay the foundation for excavating the novel targets of diagnosis and treatment in COPD.

## Introduction

Chronic obstructive pulmonary disease (COPD) is a public health challenge related to disability and mortality worldwide (Niu et al., 2022). According to the report of World Health Organization, COPD affects approximately 400 million people and has become the third main cause of mortality in the world (Lozano et al., 2012; Labaki and Rosenberg, 2020). COPD is characterized by an abnormal airway in chronic bronchitis and a substantial reduction in solid lung texture in emphysema (Rabe and Watz, 2017), eventually leading to irreversible airflow limitation and persistent respiratory symptoms (Labaki and Rosenberg, 2020). According to previous studies (Yuan et al., 2017; Hikichi et al., 2019), COPD is associated with various risk factors, including environmental deterioration, genetic factors and airway inflammation. Cigarette smoke (CS) has long been recognized as the main risk factor for the occurrence of lung disease. CS can induce persistent inflammatory responses in the airway and only a part of life-long smokers will develop COPD. In addition, some non-smokers can develop COPD, and many people diagnosed with airway restriction in childhood may develop COPD later in life (Singh et al., 2018). Accordingly, individual differences and hereditary susceptibility play an important role in the pathogenesis of COPD. However, the pathogenesis of COPD has not been clarified (Cortopassi et al., 2017; Vogelmeier et al., 2020).Therefore, this study aimed to detect the regulatory mechanisms of the ceRNA integration networks in COPD.

Over the past decades, non-coding RNAs (ncRNAs) have been considered as controversial molecules. Whereas, owing to the rapid development of high-throughput sequencing and RNA analysis techniques, ncRNAs have been suggested to participate in the pathophysiological processes of various diseases (Guttman and Rinn, 2012; Castel and Martienssen, 2013). More than 90% of human transcripts are RNA transcripts, and these transcripts are thought to be ncRNAs (Li et al., 2017). These ncRNAs can be divided into microRNAs (miRNAs), long non-coding RNAs (lncRNAs), and circular RNAs (circRNAs) (Esteller, 2011).

LncRNAs can transcribe over 200 nucleotides *via* RNA polymerase II, but do not encode proteins (Rinn and Chang, 2012). LncRNAs have been demonstrated to regulate different epigenetic, transcriptional, and post-transcriptional functions, and play an integral part in the process of lung diseases, including COPD (Kopp and Mendell, 2018; Devadoss et al., 2019). CircRNAs are another class of endogenous ncRNAs possessing covalently closed loop structures that lack 5′ caps and 3′ poly A tails (Zhang et al., 2018). For circRNAs, due to their stability and histological specificity, the mechanisms and functions are still unclear. However, increasing number of reports suggested that circRNAs could be recognized as ideal biomarkers for clinical applications (Verduci et al., 2021).

In addition, recent studies revealed a hypothesis regarding competing endogenous RNAs (ceRNAs), indicating that these RNA transcripts (including mRNA, lncRNA, pseudogenes, and circRNA) may act as natural miRNA sponges by competing for the same miRNA response elements (MERs) to regulate relevant mRNA expression induced by the ceRNA network (Salmena et al., 2011; Tay et al., 2014). On the basis of many studies, ceRNA regulation has a significant effect on the emergence and progression of COPD. For example, in COPD tissues, the low-expressed lncRNA, SNHG5, is closely involved in low-forced expiratory volume in one second (FEV1%) in patients *via* the miR-132/PTEN axis, which regulates human bronchial epithelial cell inflammation and apoptosis in COPD (Shen et al., 2020). LINC00987 can regulate lipopolysaccharide-induced apoptosis, oxidative stress, inflammation, and autophagy *via* the let-7b-5p/SIRT1 axis (Wang et al., 2020), resulting in the amelioration of COPD. CircTMEM30A is highly expressed in COPD patients with lung cancer, the circTMEM30A/hsa-miR-130a-3p axis regulates TNF-α and promotes the malignant progression of COPD with primary lung cancer (Ding and Dong, 2021). Circ-OSBPL2 promotes apoptosis, inflammation, and oxidative stress in HBECs in smoking-associated COPD through the miR-193a-5p/BRD4 axis, indicating that the potential of circ-OSBPL2 to act as a diagnostic biomarker for smoking-induced COPD (Zheng et al., 2021).

ceRNAs represent a new post-transcriptional regulatory mechanism involved in the emergence and progression of various conditions (Guttman and Rinn, 2012; Castel and Martienssen, 2013; Meng et al., 2017). Based on several investigations, the lncRNA-miRNA-mRNA and circRNA-miRNA-mRNA ceRNA networks are associated with COPD progression (Liu et al., 2022). However, only few reports have revealed the overall expression profiles of lncRNAs, circRNAs, miRNAs, mRNAs and the regulatory mechanism of pivotal lncRNA or circRNA-miRNA-mRNA ceRNA regulatory networks in smoking-induced COPD. In addition, due to the difficulty of collecting clinical samples, most bioinformatics analyses are performed with samples from public databases rather than their own clinical samples. Therefore, comprehensive analyses are needed to identify more reliable biomarkers for the occurrence and development of COPD.

In the present study, lung resection specimens from patients with COPD (COPD) (*n* = 7) and non-COPD control subjects (Normal) (*n* = 6) were chosen. Whole transcriptome sequencing (RNA sequencing [RNA-seq]) was performed to screen differentially expressed lncRNAs, circRNAs, miRNAs, and mRNAs. In addition, we constructed the lncRNA-mRNA-miRNA and circRNA-mRNA-miRNA networks through bioinformatics analysis respectively. Relied on the Kyoto Encyclopedia of Gene and Genomes pathway enrichment analysis (KEGG), Gene Ontology analysis (GO), Gene set variation analysis (GSVA), and Gene set enrichment analysis (GSEA), the crucial pathways involved in COPD were detected. To further explore the mechanism of different mRNA expression, a protein-protein (PPI) network, hub-RBP (RNA binding protein) and immune infiltration analyses were carried out. Overall, these ceRNA networks may contribute to the discovery of novel biomarkers for COPD.

## Materials and methods

### Sample collection and the ethics committee

Lung resection specimens were collected from 20 patients with solitary pneumonic tumors who underwent pneumonectomy at the Department of Thoracic Surgery, General Hospital of Ningxia Medical University between June 2020 and December 2020, in accordance with the Declaration of Helsinki. Fresh non-neoplastic lung tissue should be at least 5 cm from the neoplastic lesion. The enrolled patients were divided into two groups: In the present study, lung resection specimens from patients with COPD (COPD) (*n* = 7) and non-COPD control subjects (Normal) (*n* = 6). Patients were diagnosed based on the Global Initiative for Chronic Obstructive Lung Disease (GOLD) (Guo et al., 2018; Zhu et al., 2021). The characteristics of the participants are shown in Table 1.

**TABLE 1:** Characteristics of subjects in this study.

Characteristic	Non-COPD (Normal)	Patients with COPD(COPD)	*p*
n	6	7	
Gender (M/F), n (%)			0.005
F	5 (83.3%)	0 (0%)	
M	1 (16.7%)	7 (100%)	
Smoking history (pack-years), n (%)			0.043
0	6 (100%)	0 (0%)	
22	0 (0%)	1 (14.3%)	
25	0 (0%)	2 (28.6%)	
30	0 (0%)	1 (14.3%)	
33.75	0 (0%)	1 (14.3%)	
45	0 (0%)	1 (14.3%)	
50	0 (0%)	1 (14.3%)	
Age (Years), mean ± SD	53.83 ± 6.59	60.14 ± 11.82	0.272
BMI (kg/m2), median (IQR)	23.75 (23.38, 24.04)	25 (24.1, 25.85)	0.445
FEV1 (L), median (IQR)	3.12 (2.77, 3.4)	2.66 (2.54, 2.81)	0.198
FEV1 (% predicted), mean ± SD	117.67 ± 14.72	94.71 ± 19.93	0.040
FEV1/FVC (%), mean ± SD	80.33 ± 1.21	64.42 ± 3.69	<0.001

The study inclusion criteria for patients with COPD were as follows: (Niu et al., 2022): a post-bronchodilator forced expiratory volume in 1 s (FEV1)/forced vital capacity (FVC) rate lower than 0.70, which “verifies the existence of constant airflow restriction”; (Labaki and Rosenberg, 2020) age >40 and <80 years, current smoker with a history of cigarette smoking (more than 20 pack-years); (Lozano et al., 2012) patients with stable clinical condition that are not receiving chemotherapy or radiotherapy. The exclusion criteria for patients with COPD were as follows: (Niu et al., 2022): patients companied with lung metastasis or other organs tumors, including stomach, intestine, liver, pancreas, kidney, etc; (Labaki and Rosenberg, 2020) patients with other lung and systemic diseases, such as asthma, bronchitis, interstitial lung diseases, and cardiac, hepatic, or renal diseases; (Lozano et al., 2012) patients who inhaled or received oral glucocorticoids for 3 months before surgery and those who used biomass fuel and have a history of occupational exposure. Age- and sex-matched non-smokers without COPD and smokers with COPD served as controls.

This study was approved by the Ethics Committee of the General Hospital of Ningxia Medical University (Grant No.KYLL-2021-418). Each participant provided written informed consent.

### Whole transcriptome resequencing and data quality control

Total RNA was extracted from frozen lung tissues using Trizol Reagent (Invitrogen, Life Technologies, United States). The Qubit^®^ RNA Assay Kit for Qubit^®^ 2.0 Fluorometer (Life Technologies, CA, United States) and NanoPhotometer^®^ spectrophotometer (IMPLEN, CA, United States) were separately used to determine the concentration and purity of the total RNA. Subsequent experiments were performed with total RNA samples that met the following criteria: RNA integrity number (RIN) > 7.0 and 28S/18S ratio >1.8. First, the small RNA sequencing library was created using the NEB Next Multiplex Small RNA Library Prep Set (Illumina, San Diego CA, United States), as recommended by the manufacturer. Thereafter, a complementary DNA (cDNA) library of lncRNA was established following ribosomal RNA (rRNA) removal using the Epicenter Ribo-zero^TM^ rRNA Removal Kit (Epicenter, United States). rRNA with no residue was purified by ethanol precipitation. Sequencing libraries were produced using rRNA-depleted RNA and the NEBNext UltraTM Directional RNA Library Prep Kit for Illumina (NEB, United States), according to the manufacturer’s recommendations. Finally, all products were cleaned (AMPure XP system), and library quality was evaluated using the Agilent Bioanalyzer 2,100 system. Paired-end sequencing of individual libraries was performed on an Illumina HiSeq sequencer platform (Illumina).

Raw data (raw reads) in fastq format were initially processed using bcl2fastq or in-house Perl scripts. Clean data (clean reads) were acquired at this step by expurgating reads containing adapters, reads containing ploy-N, with 5′ adapter contaminants, without 3′ adapter or the insert tag, containing ploy A, T, G, or C, and low-quality reads from the original data. Simultaneously, the Q20, Q30, and GC content of the clean data were determined. High-quality and clean data were the basis of the entire downstream calculations.

### Identification of differentially expressed genes

The R package “Deseq2” (Love et al., 2014) was used to identify differentially expressed genes between non-smokers without COPD and smokers with COPD tissues, and these genes were called differentially expressed lncRNAs (DElncRNAs), circRNAs (DEcircRNAs), miRNAs (DEmiRNAs), and mRNAs (DEmRNAs), respectively. The screening criteria for differential genes were |log2FC| > 1 and *p*-value <.05. Genes with logFC >1 and *p*-value <.05 were identified as upregulated genes, while those with logFC <−1 and *p*-value <.05 were identified as downregulated genes. The result is visualized into volcano map and heatmap by R package ggplot2 and pheatmap respectively.

### Construction of a ceRNA regulatory network

Based on the regulatory mechanism of ceRNA networks, lncRNAs and circRNAs can act as miRNA sponges to combine miRNAs and regulate downstream target mRNAs. In this study, DEmiRNAs were employed as the center of the ceRNA network. First, target genes of DEmiRNAs were obtained using four databases: miRDB (Chen and Wang, 2020), miTarBase (Huang et al., 2020), miRanda, and TargetScan (Agarwal et al., 2015). Genes in no less than three databases were indicated as the target genes for these DEmiRNAs, and only the overlapping portions of the genes were used to construct the miRNA-mRNA relationship. The miRcode database (Jeggari et al., 2012) was used to screen the miRNA-circRNA pair mutual effects, which were then combined with the miRNA-mRNA interaction pairs to set up the DElncRNA-DEmiRNA-DEmRNA ceRNA network using Cytoscape (Shannon et al., 2003) software What’s more, the miRanda database was used to determine the connection between the DElncRNAs and DEmiRNAs. The DEcircRNA-DEmiRNA were correlated with the miRNA-mRNA interaction pairs to construct the DEcircRNA-DEmiRNA-DEmRNA ceRNA network using Cytoscape software.

### GO and KEGG enrichment analyses of DEmRNAs

GO (Gene Ontology, 2015) is a database resource for understanding the superior functions and availability of biological systems, including biological process (BP), cellular component (CC), and molecular function (MF), from large-scale molecular datasets produced using molecular-level information, especially genome sequencing and other high-throughput experimental techniques. KEGG (Kanehisa and Goto, 2000) is an extensively used database for storing information on genomes, biological pathways, diseases, and medicines. The R software package, clusterProfiler (Yu et al., 2012), was used to perform GO functional annotation and KEGG pathway enrichment analyses of DEmRNAs in the ceRNA networks. The significance levels of interest in the KEGG pathways and BPs in GO were *p*-value<0.05.

### Gene set enrichment analysis (GSEA)

GSEA (Subramanian et al., 2005) (http://software.broadinstitute.org/gsea/index.jsp) is a genome-wide expression profile chip data analysis method for identifying functional enrichment through a comparison of genes and predefined gene sets. A gene set is a set of genes that share localization, pathways, functions, or other characteristics. GSEA can be used to assess related pathways and molecular mechanisms in smokers with COPD. We obtained the “hall.v7.2. symbols.gm” gene set in the MSigDB (Liberzon et al., 2015) database (v7.5.1) and performed GSEA on the differentially expressed mRNAs using the R package for GSEA. A false discovery rate (FDR) <.25 was considered to indicate obvious enrichment.

### Gene set variation analysis (GSVA)

The R package, GSVA (Hanzelmann et al., 2013), was used to determine the scores of the relevant pathways underpinned by the gene expression matrix of every sample using single-sample gene set enrichment analysis (ssGSEA), and differentially screened many functions (or pathways) using the limma package (Ritchie et al., 2015).

### Construction and analysis of the protein-protein interaction (PPI) network

PPI analysis of known differentially expressed genes and predicted PPIs was performed using the STRING database (Szklarczyk et al., 2015) (http://string-db.org; version11.5).

The Cytoscape software (version 3.6.1) Network Analyzer was used to calculate the node degree. cytoHubba (Chin et al., 2014) is a Cytoscape plug-in used to study the hub genes of the PPI network.

Combined PPI pairs with a confidence value of 0.9 were retrieved, and data from the PPI table were inputted into the Cytoscape software to create a visual PPI network. By employing the MCODE (Version 2.0.0) plug-in in the software to select hub modules in the PPI network, the GOSemSim (Yu et al., 2010) package was applied to conduct a Friends analysis on the first two core clusters. The cytoHubba plugin was also used to study hub genes in the PPI network.

### Quantitative real-time PCR (qRT-PCR) for identification of hub genes

Total RNA was isolated from non-smokers without COPD (Normal) (*n* = 5) and smokers with COPD (COPD) (*n* = 5) using TRIzol reagent (Invitrogen, Life Technologies, United States), and cDNA was derived using the RevertAid First Strand cDNA Synthesis Kit (Thermo Scientific, United States). qRT-PCR was conducted using the CFX Connect Real-time PCR system (Bio-RAD, United States) and the TB Green^®^ Premix Ex Taq™ II (Tli RNaseH Plus) kit (Takara Bio, Japan), according to the instructions. The housekeeping gene, *GAPDH*, was used for normalization. All primer sequences are shown in Supplementary Table S1. Data represent the average of three independent replicates.

### Immune infiltration analysis

CIBERSORTx (Chen et al., 2018) deconvolves the transcriptome expression matrix, which is based on the theory of linear support vector regression, to predict the composition and richness of immune cells in mixed cells. The gene expression matrix data were uploaded to CIBERSORTx, and combined with the LM22 eigengene matrix. Samples with *p* < .05 were filtered, and the immune cell infiltration matrix was obtained. The R language ggplot2 package was used to draw histograms to represent the distribution of 22 types of immune cell infiltration in every sample. For the two study groups, a boxplot was generated to demonstrate the relative abundance of immune cell infiltration. The correlation between the expression of key genes and the content of various types of immune cells was also analyzed.

### Construction of the RBP-gene and TF-target gene

RNA binding proteins (RBPs) play a vital role in gene regulation. Currently, most RNAs bind to proteins to form RNA-protein complexes, except a few RNAs that can function as ribozymes alone. RBPs play a key role in the regulation of life activities, such as RNA synthesis, alternative splicing, modification, transportation, and translation. Consequently, analyzing the interaction between RNA and protein is key for evaluating the function of RNA. The starBase (Li et al., 2014) database is based on high-throughput CLIP-Seq and degradome experimental data. The database contains miRNA-ncRNA, miRNA-mRNA, RBP-RNA, and RNA-RNA data. RBPs can recognize special RNA binding domains and interact with RNA in cells, which belong to a type of post-transcriptional protein, and can participate in the control of RNA splicing, transport, sequence editing, intracellular localization, and translation. In this study, the hub-RBP network was constructed using the starBase database (https://starbase.sysu.edu.cn/) and visualized using Cytoscape software.

Transcription factors (TFs) control gene expression by interacting with target genes during the post-transcriptional stage. To analyze the regulatory effect of TFs on hub genes, specific binding of TFs to gene regulatory regions is an important approach for the regulation of gene expression. TF prediction was performed using the JASPAR database (JASPAR 2018) (Vlieghe et al., 2006) and TFBSTools software (3.3.2) (Tan and Lenhard, 2016), and the binding sites of TFs within the region 2,000 bp upstream of the start site of each lncRNA and 500 bp downstream, direction and scoring results were provided. The hub-TF interaction networks were visualized using Cytoscape software.

### Statistical analysis

All calculations and statistical analyses were carried out at https://www.r-project.org/ (version 4.0.2). For the comparison of two groups of continuous variables, an independent Student’s t-test was used to estimate the statistics of normally distributed variables, and the Mann-Whitney U test (Wilcoxon rank sum test) was used to analyze the differences between non-normally distributed variables. All statistical *p* values were two-sided, and *p* < .05 was considered statistically significant.

## Results

### Identification of DEGs in COPD

**FIGURE 1:**
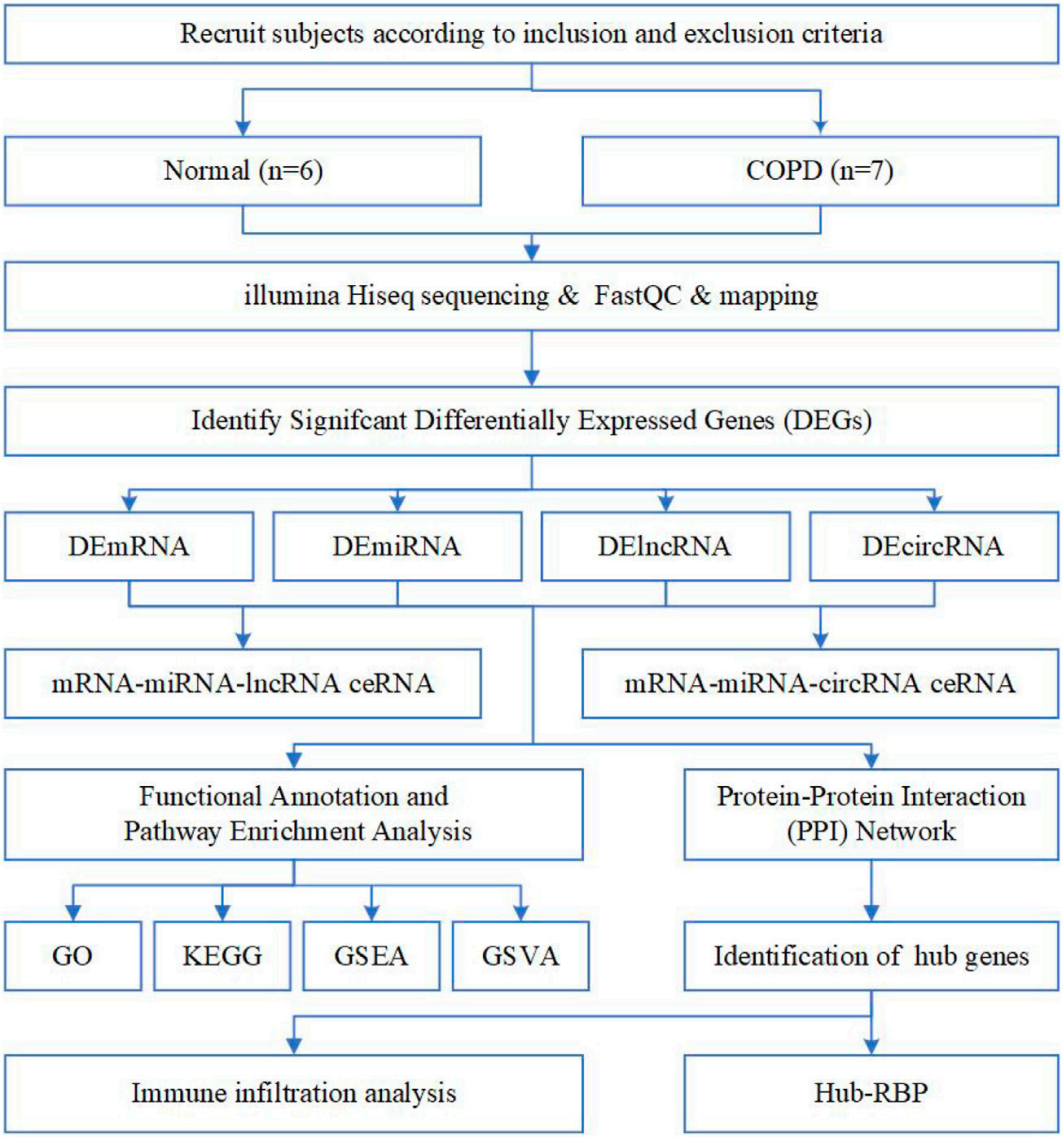
Flow chart of the overall analysis to explore the biological characteristics of COPD by bioinformatics methods.

A total of 13 individuals participated in the study, including 6 in the normal and 7 in the COPD group (Table 1). The analysis strategy and procedure used in this study are illustrated in Figure 1.

**FIGURE 2:**
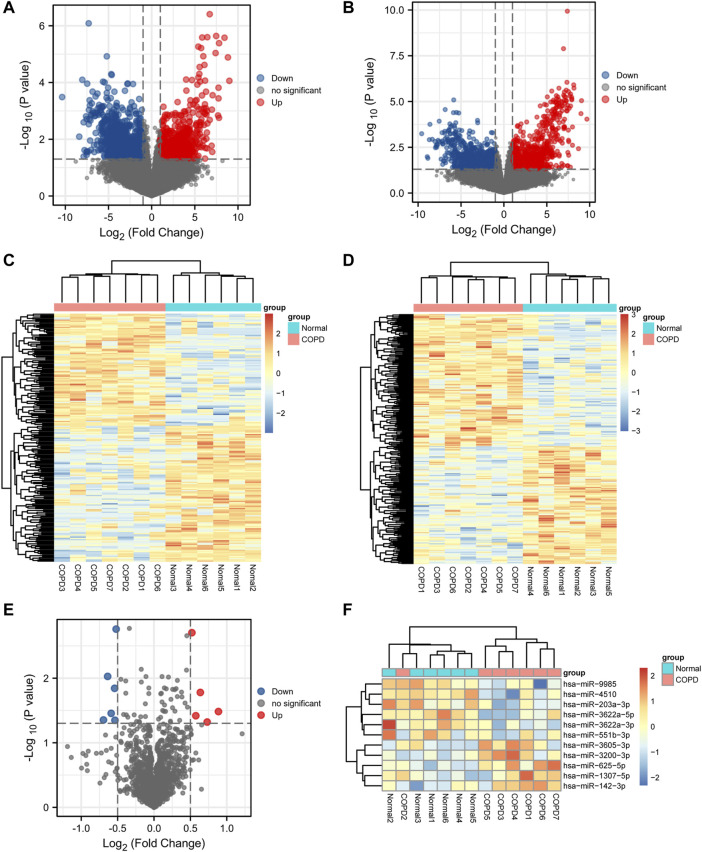
Differential expression analysis. **(A,B,E)**, Volcano plot of differentially expressed mRNA, lncRNA, and miRNA analysis. **(C,D,F)**, Heatmap presentation of differential mRNAs, lncRNAs, and miRNAs.

A total of 1,796 DEmRNAs were identified, of which 796 were upregulated and 1,000 were downregulated. A total of 2,207 DElncRNAs were identified, of which 1,245 were upregulated and 962 were downregulated. Finally, 11 DEmiRNAs were identified, among which 5 were upregulated and 6 were downregulated. Volcano plots (Figures 2A, B, E) and heat maps (Figures 2C, D, F) of the DEGs were generated to visualize the difference between the COPD group and the normal group.

### Construction and analysis of the ceRNA network

**FIGURE 3:**
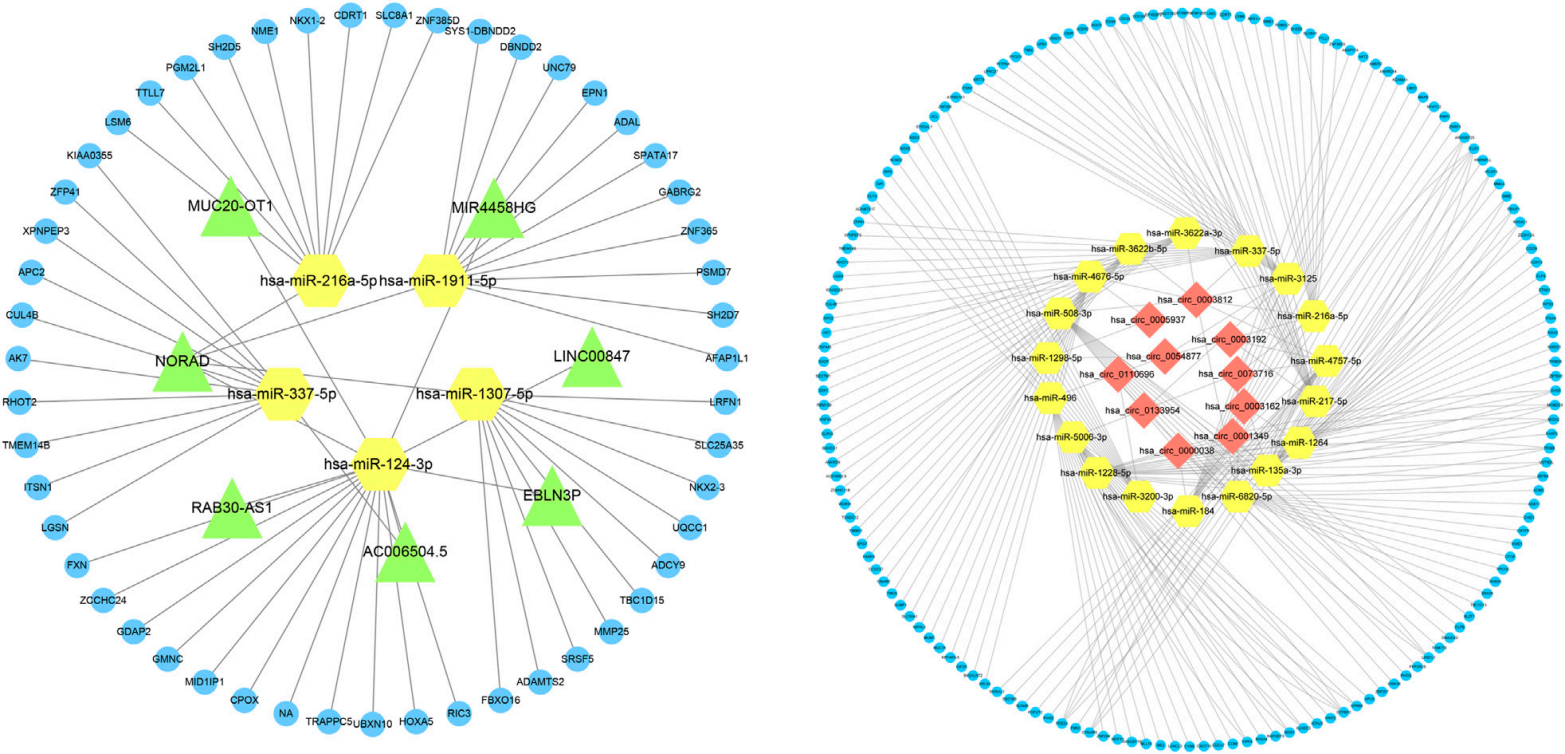
Interaction network of mRNA-miRNA-lncRNA and mRNA-miRNA-circRNA. The interaction network of differentially expressed mRNA-miRNA-lncRNA and mRNA-miRNA-circRNA, in which the yellow node is miRNA, the green node is lncRNA, the blue node is mRNA, and the red node is circRNA.

Based on the expression profiles of miRNAs, lncRNAs, and mRNAs for COPD patients and normal participants, we established a lncRNA-miRNA-mRNA ceRNA network, which contained a total of 5 miRNA, 51 mRNA and 7 lncRNA nodes (Figures 1–3). Furthermore, a circRNA-miRNA-mRNA ceRNA network based on the expression profiles of miRNA, circRNA, and mRNA in COPD patients and normal participants was constructed. The ceRNA network contained 19 miRNA, 169 mRNA, and 10 circRNA nodes (Figures 2, 3).

### PPI network and hub gene identification

**FIGURE 4:**
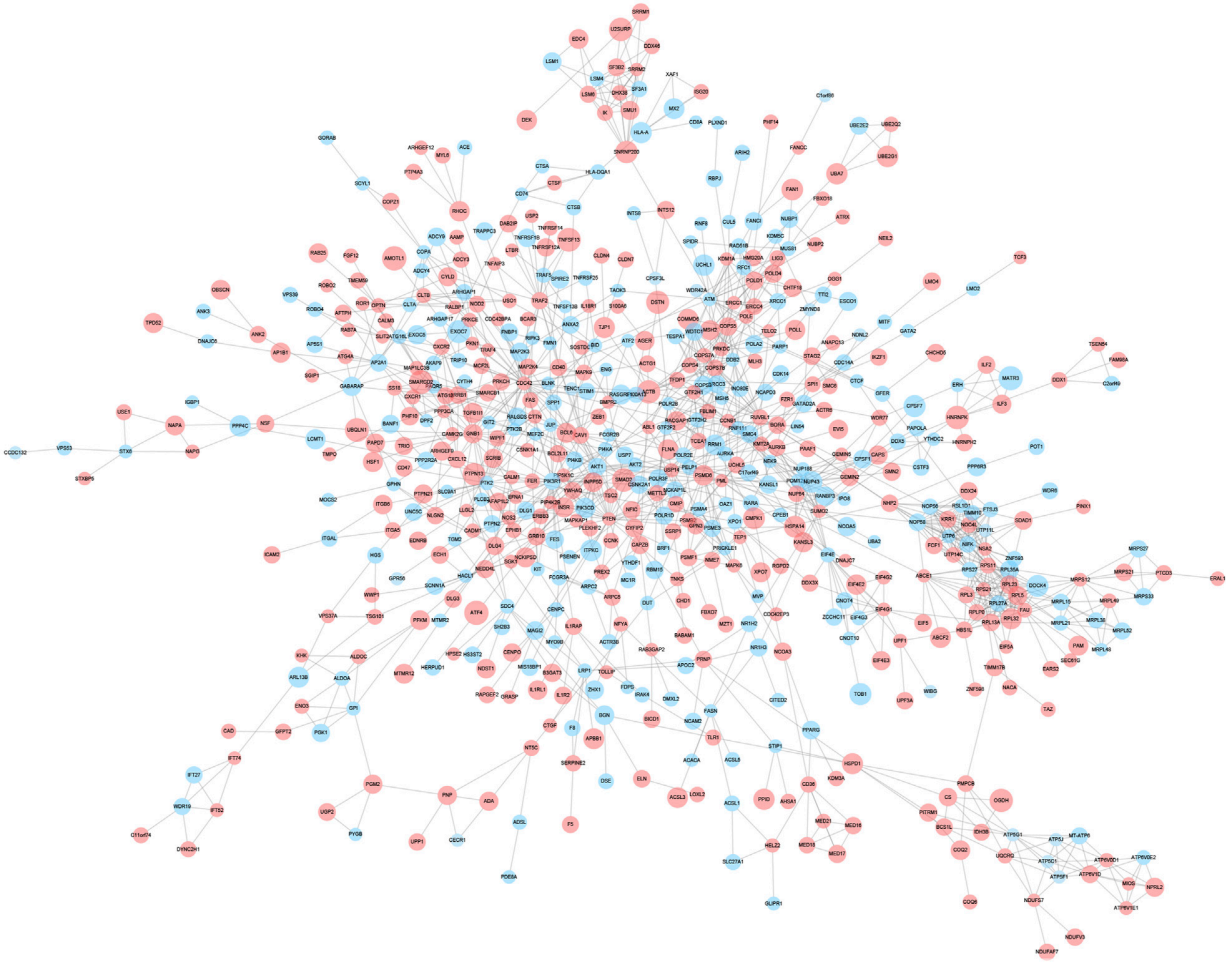
Protein-protein interaction network. Protein-protein interaction analysis of DEGs was performed using STRING data, and the interaction relationship was visualized. The larger the circle,the higher the fold of differential expression. Blue indicates genes with down-regulated expression and red indicates genes with up-regulated expression.

**FIGURE 5:**
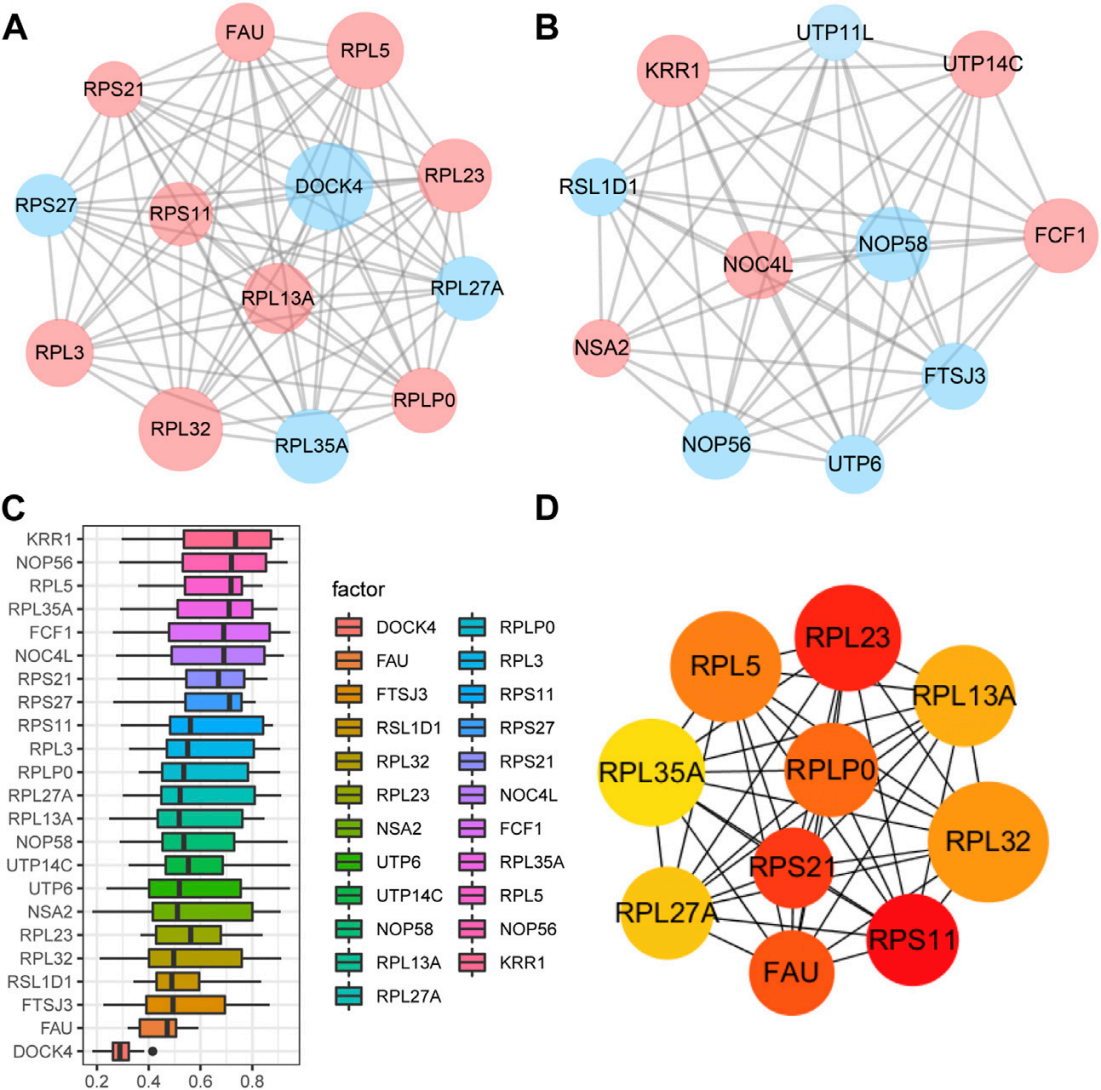
Key analysis of differences between COPD and Normal. **(A,B)**, MCODE plugins selected the first two hub modules in the PPI network, where blue indicates up-regulated genes and red indicates down-regulated genes. **(C)**, Friends analysis of genes in the first two clusters was performed using the GOSemSim package, with similarity scores on the abscissa and gene names on the ordinate, where genes with higher scores were more important. **(D)**, For the top ten Hub genes in the PPI network analyzed by the CytoHubba plug-in, darker colors indicate higher MCC scores.

**FIGURE 6:**
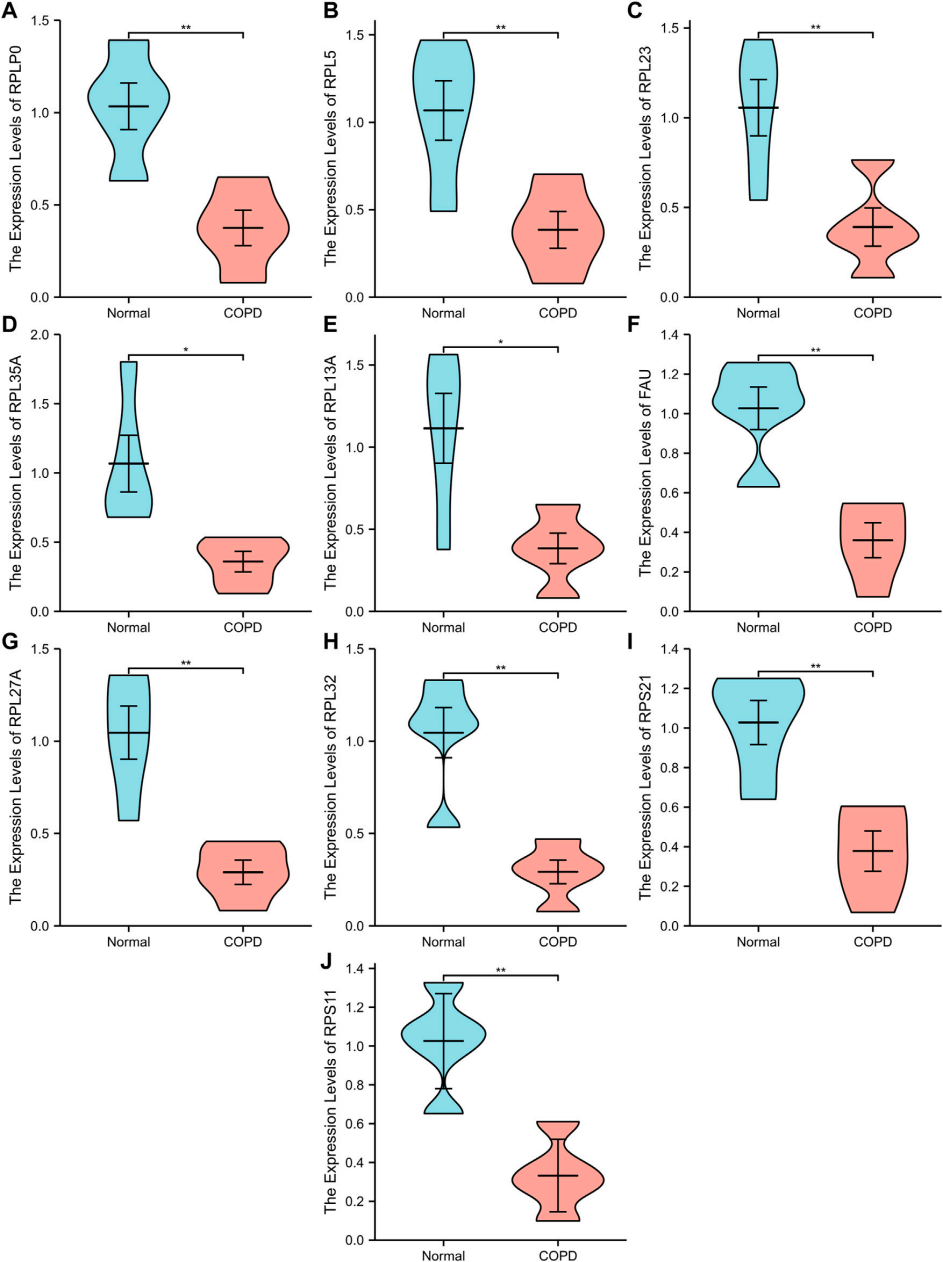
Ten differential expression Hub genes. **(A-J)**, RT‐qPCR was used to verify the hub genes between COPD group and Normal group. “**” *p*<0.01.

A PPI network associated with DEmRNAs was constructed through the STRING database, visualizing the interaction relationship, which included 616 nodes and 1,424 edges. (Figure 4). The first two hub modules in the PPI network, Cluster1 (MCODE score = 12.667) and Cluster2 (MCODE score = 10.6) were selected using MCODE in the software (Figures 5A, B). Cluster1 contains 13 genes, of which 4 genes expression up-regulation were *RPS27, DOCK4, RPL27A, RPL35A*, the 9 genes expression down-regulated were *RPS11, RPL23, RPL3, RPS21, FAU, RPLP0, RPL5, RPL13A*, and *RPL32* (Figure 5A). Cluster2 contains 10 genes, of which 5 were up-regulated, namely *NOP58, NOP56, FTSJ3, UTP6, RSL1D1*, and 5 were down-regulated, namely *KRR1, NSA2, FCF1, NOC4L, UTP14C* (Figure 5B). We further used the GOSemSim package to perform Friends analysis on the genes in the first two hubs, and the results suggested that the *KRR1* was more important (Figure 5C). We then used the cytoHubba plugin to analyze the MCC algorithm to select the top 10 genes, namely *RPS21, RPL32, RPL35A, FAU, RPLP0, RPS11, RPL27A, RPL23, RPL5, RPL13A* as core genes (Figure 5D). We verified the mRNA levels of the top 10 hub genes in the COPD group, and we found that expression of 8 hub genes (*RPLP0, RPL5, RPL32, RPL13A, FAU, RPL32, RPS21* and *RPS11*) was significantly downregulated in COPD tissues compared to the normal tissue consisted with the prediction results (Figures 6A–J).

### Construction of the RBP-genes and TF-target gene network

**FIGURE 7:**
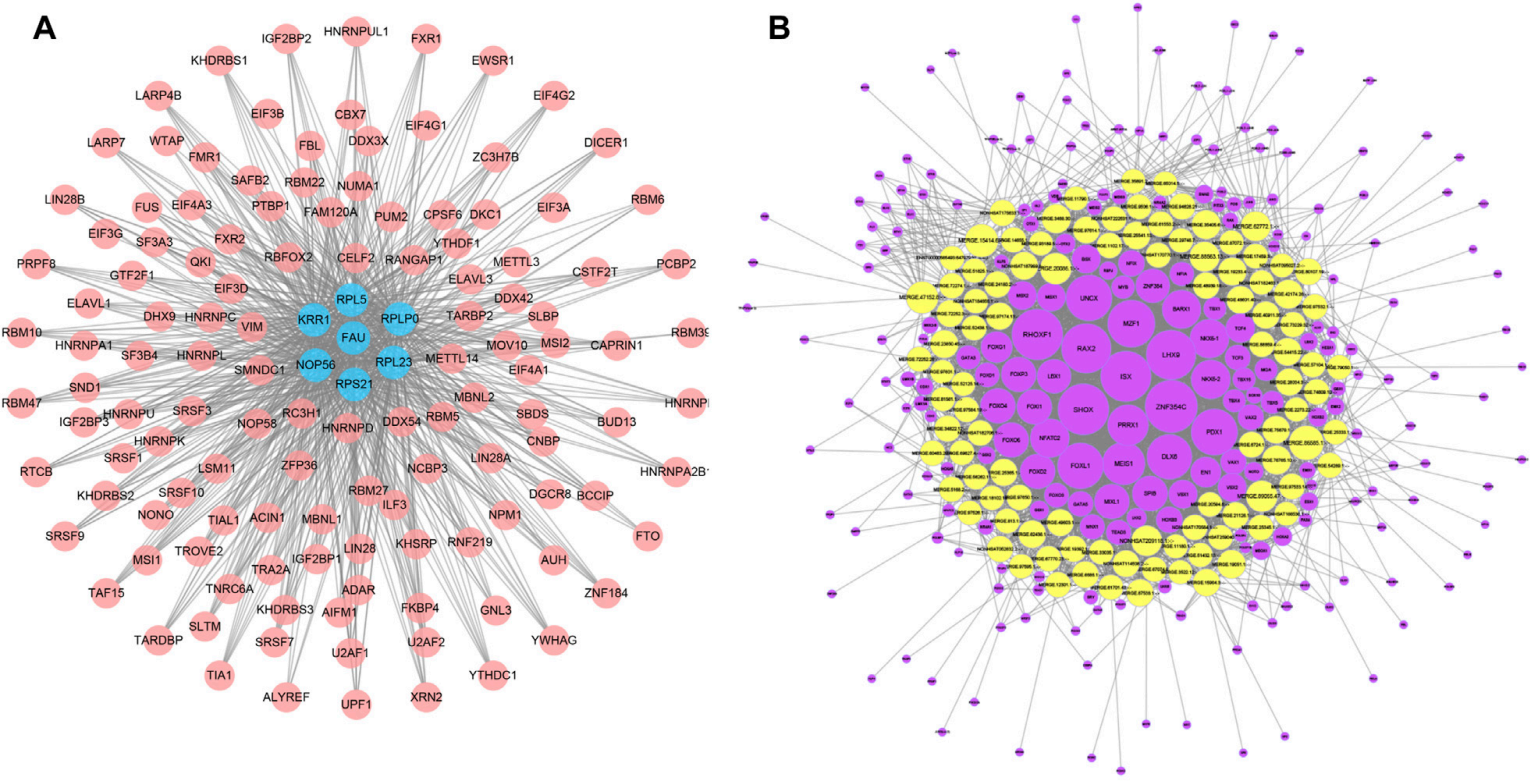
mRNA-RBP and TF-lncRNA networks. **(A)**, Diagram of the interaction network between key mRNA genes and RBPS, where pink circle node represent RBPS and blue nodes represent the corresponding mRNAs. **(B)**, network diagram of the interaction between lncRNA and TF transcription factors, where the yellow is the differential lncRNA and the purple node represents the TF.

We applied starBase database to construct a mRNA-RBP network, which comprised 7 mRNAs (FAU, RPS21, KRR1, NOP56, RPL5, RPL23, RPLP0) and 127 RBPs, of which RPL5 interacted with 118 RBPs, RPLP0 with 106 RBPs, RPL23 with 115 RBPs, FAU with 90 RBPs, RPS21 with RBPs, KRR1 with 103 RBPs and NOP56 with 114 RBPs (Figure 7A). We subsequently constructed a TF-lncRNA network consisting of 100 lncRNAs and 231 TFs using JASPAR database and TFBSTools software (Figure 7B). The top 10 TFs were ZNF354C, RHOXF1, SHOX, ISX, LHX9, RAX2, MZF1, PDX1, FOXL1, UNCX. Among them, ZNF354C was the transcription factor that interacted with the most lncRNAs (97 lncRNAs) in the TF-lncRNA network. (Figure 7B).

### Functional enrichment analysis of DEmRNAs

**FIGURE 8:**
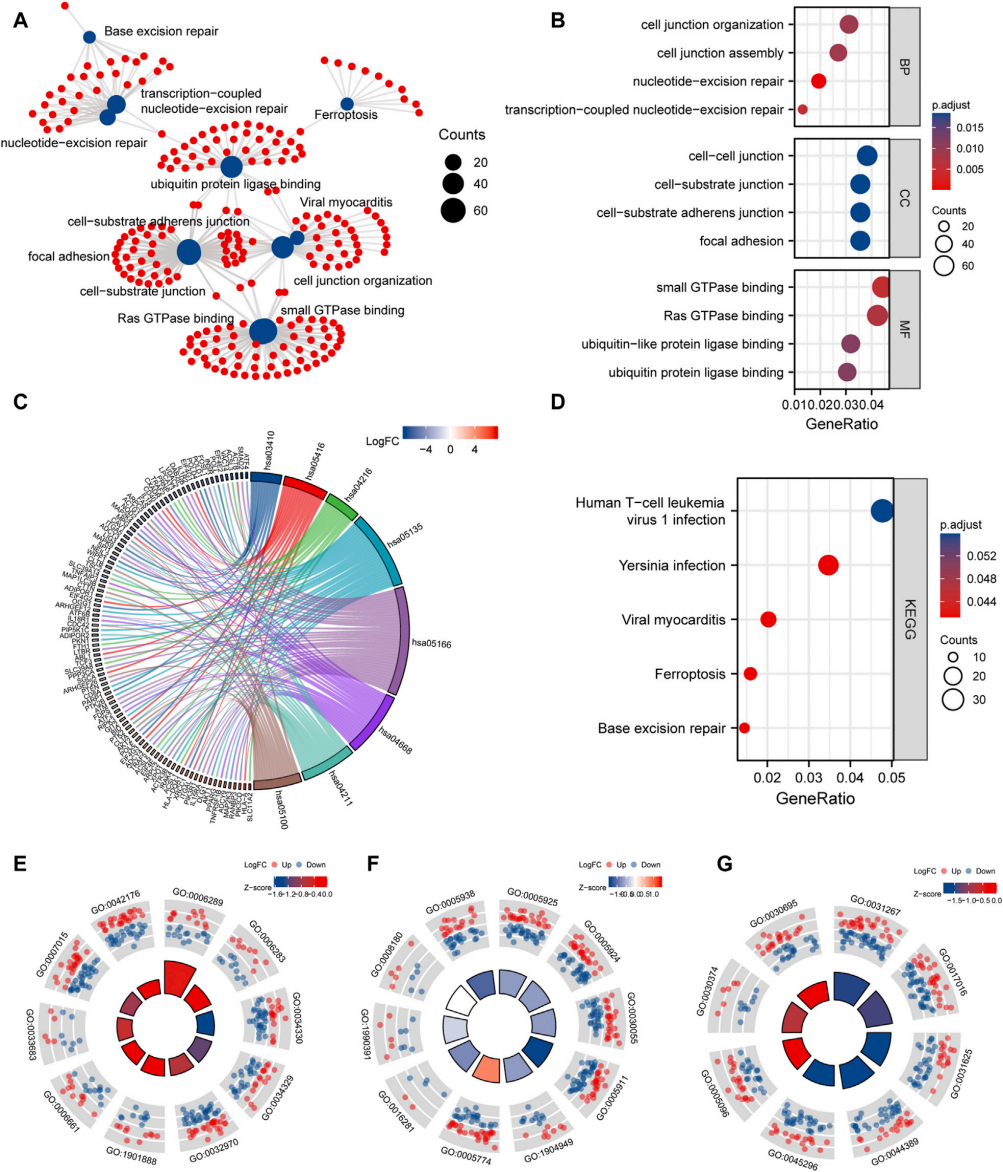
GO and KEGG enrichment analysis. **(A)**, network diagram of GO and KEGG functional enrichment of differential mRNAs. **(B)**, GO analysis dot plot of differential mRNA, abscissa is -log (p.adjust), ordinate is GO terms. **(C)**, Chordal diagram of KEGG analysis. The quadrangle corresponding to the differentially expressed genes on the left shows downregulated expression in blue and upregulated expression in red. **(D)**, KEGG enrichment Pathway map of differential genes, the horizontal axis is gene ratio, the vertical axis is Pathway name, the node size represents the number of genes enriched in the pathway, and the node color represents p.value. **(E–G)**, are the visualization results of functional enrichment of BP, CC and MF, respectively. The outer circle is the GO terms, the red dot indicates the up-regulated genes, the blue dot indicates the down-regulated genes, the quadrate color indicates the z-score of GO terms, and the blue indicates that the z-score is negative, which means that the corresponding GO terms are more likely to be inhibited. Red indicates that the z-score is positive and is more likely to be activated in the corresponding GO terms.

To study the relationship between DEmRNAs and BPs, MFs, CCs, biological pathways, and diseases, we first performed functional enrichment analysis of DEmRNAs (Figures 8A–G; Supplementary Tables S2, S3). DEmRNAs were the most abundant in BPs, such as nucleotide-excision repair, transcription-coupled nucleotide-excision repair, cell junction organization, cell junction assembly, and control of actin filament-based process (Figure 8E). Further, the DEmRNAs were enriched in CCs, such as focal adhesion, cell-substrate adherens junction, cell-substrate junction, cell-cell junction, ATPase complex (Figure 8F). Small GTPase binding, Ras GTPase binding, ubiquitin protein ligase binding, ubiquitin-like protein ligase binding, cadherin binding, and other MFs were identified (Figure 8G).

Next, KEGG pathway enrichment analysis was performed on DEmRNAs. Based on the results, the DEmRNAs were abundant in biological pathways, such as base excision repair, ferroptosis, *Yersinia* infection, and human T-cell leukemia virus 1 infection (Figure 8D).

### GSEA and GSVA

**FIGURE 9:**
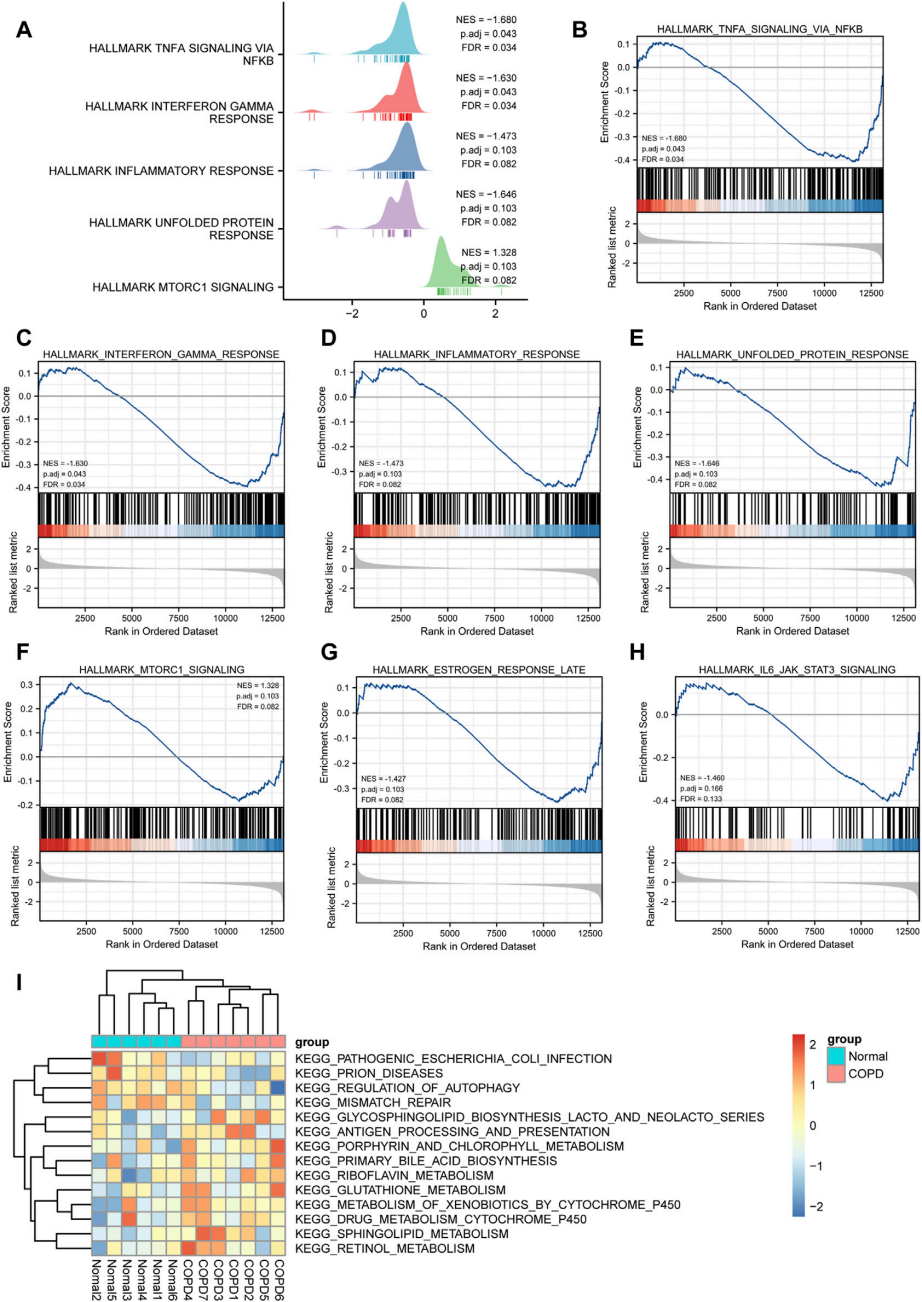
GSEA and GSVA analysis. **(A)**, GSEA analysis showed five main biological functions. **(B-H)** and GSEA analysis suggested that themain enriched pathways in the case group COPD group. **(I)**, Heat map presentation of GSVA analysis.

To determine the impact of gene expression levels on disease, GSEA was performed to analyze the relationship between gene expression and the BPs, CCs, and MFs. GSEA revealed that the most significantly enriched gene sets were negatively correlated with the COPD group, which included the TNF-α signaling *via* NF-κB, interferon gamma response, inflammatory response, unfolded protein response, mtorc1 signaling, estrogen response late, IL6/JAK/STAT3 signaling. Interestingly, these phenotype characteristics are thought to be associated with the progressions of COPD (Figures 9A–H; Supplementary Table S4).

The results of GSVA suggested that COPD group was mainly enriched in KEGG pathogenic *Escherichia coli* infection, prion diseases, regulation of autophagy, mismatch repair, glycosphingolipid biosynthesis lacto and neolacto series, antigen processing and presentation, porphyrin and chlorophyll metabolism, primary bile acid biosynthesis, riboflavin metabolism, glutathione metabolism, metabolism of xenobiotics by cytochrome p450, drug metabolism cytochrome p450, sphingolipid metabolism, retinol metabolism, and other biologically related functions and signaling pathways (Figure 9I).

### Immune infiltration analysis

**FIGURE 10:**
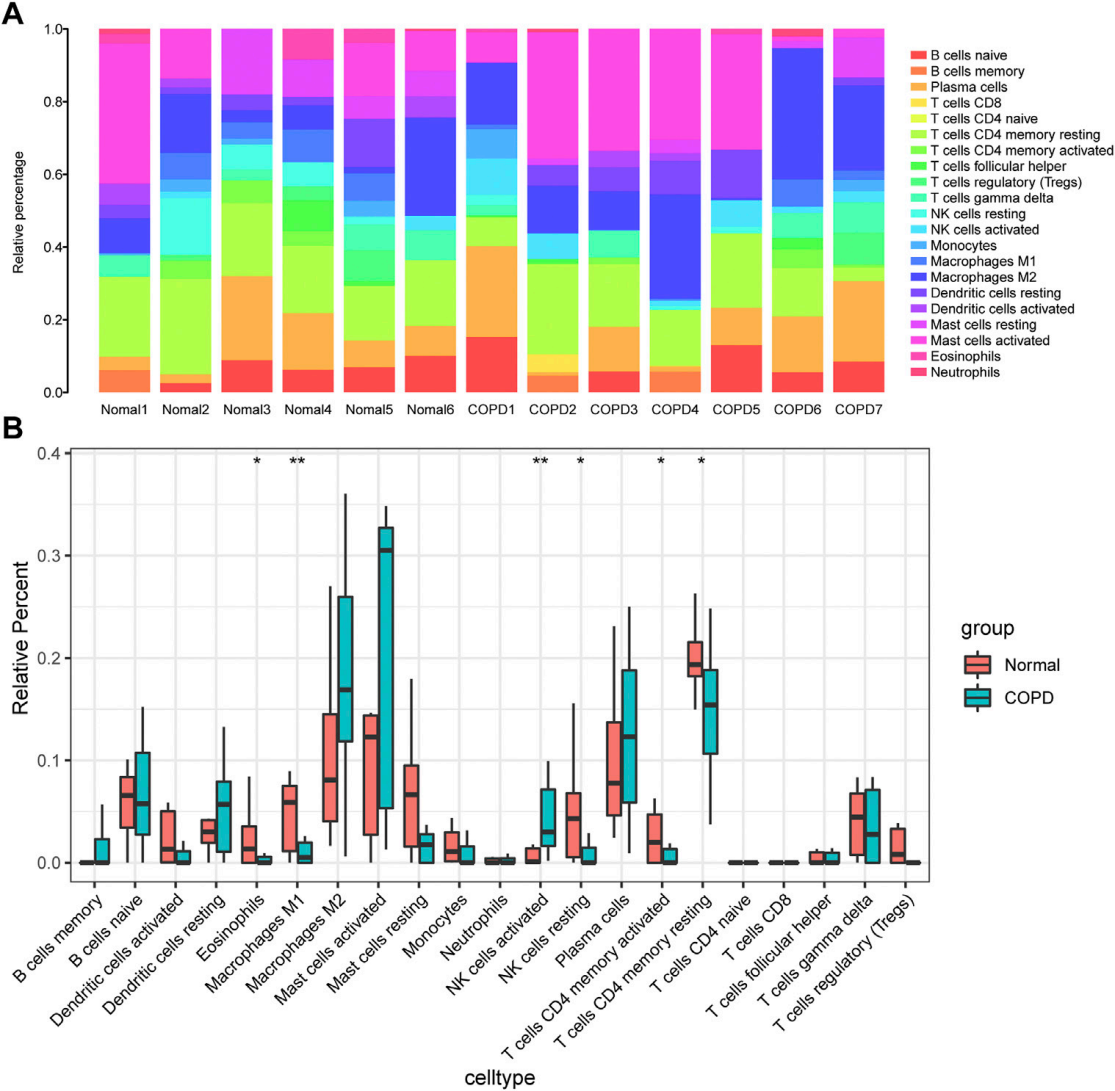
Analysis of immune infiltration. **(A)**, component analysis of immune cells in COPD and control samples; **(B)**, Differential analysis of the composition of various immune cells in the samples of COPD group and control group. The meanings represented by different asterisks explain significant differences. * indicates that the difference is statistically significant, “*” *p* < 0.05; “**” *p* < 0.01.

In this study, the gene expression matrix data were analyzed for immune cell infiltration, and filtered an immune cell infiltration matrix (*p* < .05) that revealed the distribution of immune cells (Figure 10A). The differences in immune cell infiltration between the normal group and COPD group were analyzed, the proportions of Eosinophils, M1 Macrophages, activated memory CD4^+^ T cells, resting NK cells and resting memory CD4^+^ T cells were higher in normal group. In addition, activated NK cells had a higher proportion of infiltration in COPD group (Figure 10B).

**FIGURE 11:**
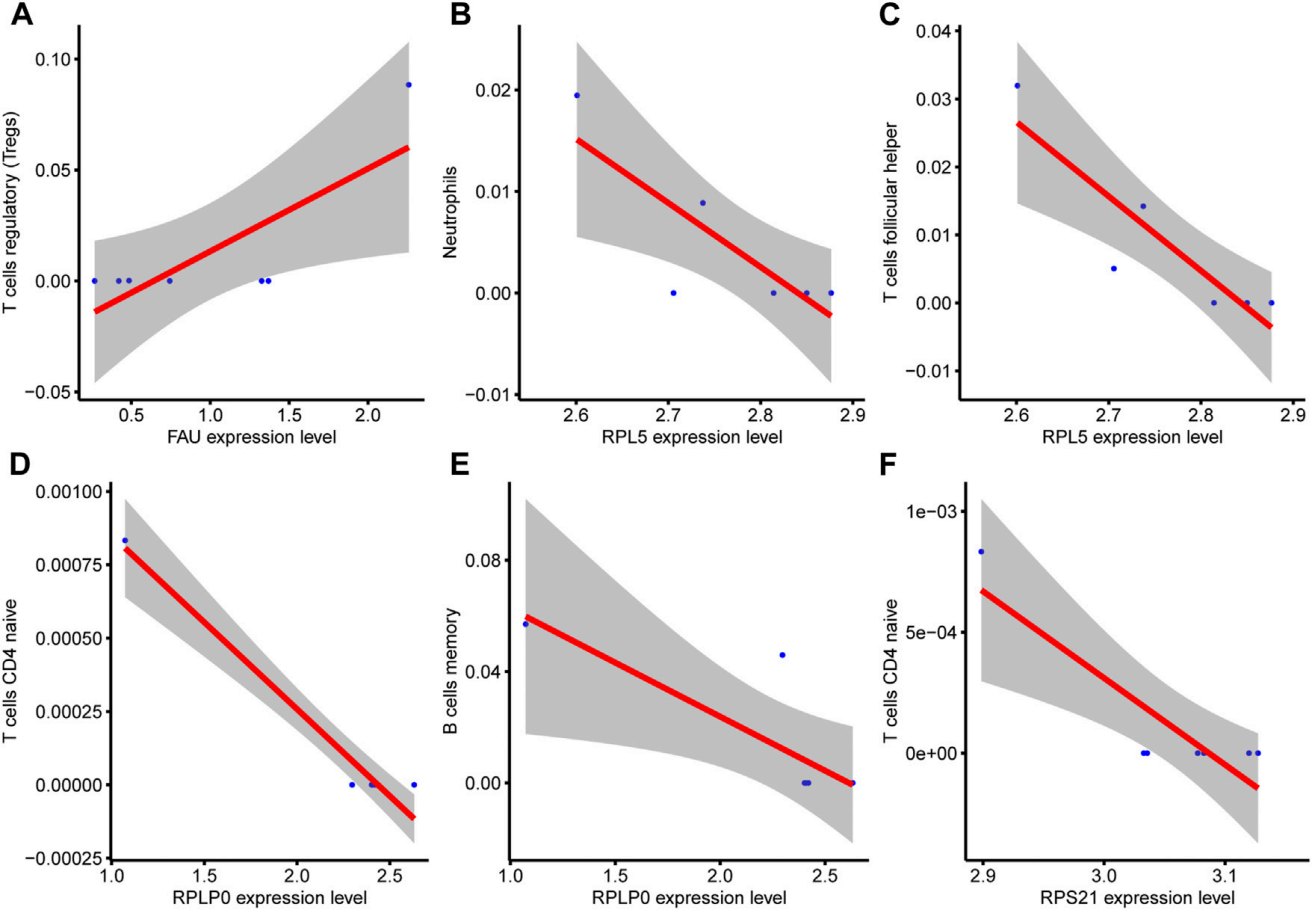
Correlation analysis between hub genes and immune cells. **(A)**, There was a positive correlation between FAU expression and immune cells T cells regulatory (Tregs) in COPD group. **(B)**, RPL5 gene expression was negatively correlated with Neutrophils **(C)**, RPL5 gene expression was negatively correlated with T cell follicular helper. **(D)**, RPLP0 gene expression was negatively correlated with the immune cell component CD4 naive T cells. **(E)**, RPL10 gene expression was negatively correlated with B cell memory. **(F)** and RPS21 gene expression were negatively correlated with CD4 naive T cells.

At the same time, the correlation between the infiltration of various immune cells and hub genes in the COPD group was analyzed (Figures 11A–F). There was a positive correlation between FAU gene expression and T cells regulatory (Tregs) in the COPD group (Figure 11A). RPL5 was negatively correlated with Neutrophils (Figure 11B). RPL5 was negatively correlated with T cells follicular helper (Figure 11C). RPLP0 was negatively correlated with T cells CD4 naive (Figure 11D). RPL10 was negatively correlated with B-cell memory (Figure 11E) and RPS21 was negatively correlated with CD4 naive T cells (Figure 11F).

## Discussion

COPD is a heterogeneous disease in which chronic bronchiolitis and emphysema are the most prominent phenotypes and remain the leading causes of death worldwide (Mirza et al., 2018). With the evolution of the high-throughput sequencing technology and bioinformatics analysis, the ceRNA network hypothesis may illustrates the occurrence and progression of disease partially. Despite an increasing number of studies on ceRNA networks, it was not been fully elaborated about the molecular mechanisms of COPD (Salmena et al., 2011; Gong et al., 2020; Chen et al., 2022). In the present study, we utilized the whole transcriptome sequencing analysis of two groups (seven patients with COPD and six non-COPD control subjects), screened out 1,796 DEmRNAs (796 upregulated and 1,000 downregulated), 2,207 DElncRNAs (1,245 upregulated and 963 downregulated), and 11 DEmiRNA (five upregulated and six downregulated).

To date, the functions of most lncRNAs and circRNAs remain unclear. Consequently, the construction of a ceRNA network of lncRNAs/circRNA-miRNAs-mRNAs could provide help for the prediction of the functions of lncRNAs/circRNAs. According to the ceRNA co-expression network, 7 lncRNA-5miRNA-51mRNA and 10circRNA-19miRNA-169mRNA ceRNA networks were selected for further investigation respectively. LncRNAs regulate gene expression at different levels, including epigenetic, transcriptional, and post-transcriptional, which can act as miRNA sponges and interfere with miRNA-mediated degradation of target mRNA (Quinn and Chang, 2016; Kopp and Mendell, 2018). For example, the lncRNA, NORAD, is upregulated in non-small cell lung cancer (NSCLC) and accelerates the progression of NSCLC by enhancing tumor cell proliferation by targeting the miRNA-455/CDK14 axis (Wang et al., 2021). Similarly, the expression of the NORAD was notably increased in cancer tissues and cells compared with that in normal tissues and cells in NSCLC, which regulates the proliferation, migration, and invasion capabilities of NSCLC cells by targeting the miR-520a-3p/PI3k/Akt/mTOR signaling pathways (Wan et al., 2020). Wang et al. revealed that the lncRNA, EBLN3P, was upregulated in lung adenocarcinoma cell lines (A549 and NCI-H23), inhibiting A549 cell viability and promoting apoptosis *via* the miR-655-3p/Bcl-2 axis (Wang and Yin, 2022). CircRNA is a covalently closed loop-like structure that is highly specific to the eukaryotic transcriptome and can be used as a microRNA sponge, a splicer, and for transcribed gene expression (Qu et al., 2015). Subsequently, out of the 10 DEcircRNAs from circRNA-miRNA-mRNA ceRNA network in this study, only 1 DEcircRNAs had reported to be associated with lung diseases. Yang et al. reported that hsa_circ_0003162 is significantly down-regulated in lung adenocarcinoma, indicating that it may be involved in the progression of lung adenocarcinoma (Liu et al., 2021). However, none of the other 9 circRNAs have been reported, which need further *in vitro* and *in vivo* experiments, might serve as novel potential biomarkers for COPD. Thus, these ceRNA networks indicate that our bioinformatics approach can effectively identify the potential functions of lncRNAs and circRNAs. To sum up, our results are consistent with most current studies focusing on lncRNA or circRNA-miRNA pairs and hopefully provide useful information for future research on COPD.

In our research, the STRING database was used to generate PPI with DEmRNAs, which were keeping a high degree of consistency with confirmatory experiment. The mRNAs including RPL5, RPL11, RPL27A and RPL32 are significantly informative. As far as we know, most ribosome proteins (RPs) are connected with cell growth, proliferation, differentiation and apoptosis. Liao et al. reported that ribosomal protein L5 (RPL5) and ribosomal protein L11 (RPL11) synergistically guide RNA-induced silencing complexes (RISCs) into c-Myc mRNA and degrade their mRNA, thereby inhibiting the activity of c-Myc in human lung adenocarcinoma cells (H1299) (Liao et al., 2013). Park et al. reported that under stimulation, RPL5 further inhibits the upsurge and promotes apoptosis of NSCLC cells by inhibiting c-Myc (Park et al., 2021). Xie et al. found that silencing of RPL32 causes RPL5 and RPL11 to be transferred from the nucleus to the nucleoplasm, leading to the accumulation of p53 and inhibition of lung cancer proliferation (Xie et al., 2020). The expression of RPS27a in LUAD was also found to be upregulated, suggesting that the expression of RPS27a may be related to LUAD progression and poor prognosis (Li et al., 2022). These results are consistent with our research. Thus, we speculated that RPL5, RPL11, RPL27A and RPL32 might have influence on the pathogenesis of COPD by regulating the above phenotypes, which expected to be potential biomarkers for COPD. Overall, comprehensive analysis of hub genes in COPD may offer new perspectives on the pathogenesis of COPD.

Furthermore, the biological function of DEmRNAs was identified grounded on GO annotation and KEGG pathway enrichment analysis. The nucleotide-excision repair, base excision repair, Ferroptosis, *Yersinia* infection, Human T-cell leukemia virus 1 infection were related to the pathophysiologic mechanism of COPD. Then, we performed GSEA and GSVA analyses to further elucidate the underlying mechanisms. The GSVA heatmap result revealed that the activity of glutathione metabolism, metabolism of xenobiotics by cytochrome p450, drug metabolism cytochrome p450 was enhanced in smokers with COPD samples, whereas regulation of autophagy was impeded. GSEA result revealed relatively high enrichment of TNF-α *via* NF-κB, interferon gamma response, inflammatory response, IL6/JAK/STAT3 signaling pathways in smokers with COPD patients. Among which TNF-α *via* NF-κB play a significant role in COPD pathology. TNF-α is an important pro-inflammatory cytokine produced by different immune inflammatory cells (such as epithelial cells) in response to stimulation. In COPD, TNF-α recruit inflammatory cells producing inflammatory mediators, which activated airway inflammation response caused airway oxidation and hyperreactivity. Chen et al. suggested that TNF-α stimulates interleukin-6 (IL-6) and interleukin-8 (IL-8) generation, activating the nuclear factor-κB (NF-κB) pathway by the degradation of IκB-α and the phosphorylation and nuclear migration of NF-κB p65, highlight the role of TNF-α in the pathogenesis of chronic inflammation, suggesting that TNF-α may be a promising target for the treatment of airway inflammatory diseases especially COPD (Brightling et al., 2008; Herfs et al., 2012; Chen et al., 2020), which were consistented with our study. The JAK/STAT pathway is activated by a variety of pro-inflammatory cytokines, such as IL-6, IL-11, and IL-13, which are upregulated in different lung diseases (Montero et al., 2021). Eskiler G et al. revealed that IL-6-mediated Janus kinase (JAK)/signal sensor and transcriptional activator 3 (STAT3) pathways are indispensable in cancer cachexia, such as lung cancer *via* the induction of a systemic inflammatory response. Johnson et al. revealed that the IL6/JAK/STAT3 pathway is abnormally activated in many types of cancer, which is often associated with poor clinical prognosis (Johnson et al., 2018). All these above views indicated that TNF-α *via* NF-κB and IL6/JAK/STAT3 signaling pathways were implicated with pathogenesis of COPD.

Despite this, our study had some limitations. First, owing to the small sample size used in this study, it is impossible to comprehensively summarize the COPD transcriptome. Thus, the sample size and male to female ratio should be expanded for further analysis. Second, due to the limitations of the current environment, although we are also interested in the comparison for ceRNA networks between patients with lung tumor vs. no tumor COPD patients, However, no similar samples have been collected, and no similar public transcriptome data of lung tumor vs. no tumor COPD patients have been searched in public databases, so it cannot be done at present. Our next step is to collect such samples as much as possible and then perform whole-transcriptome sequencing. Third, because smoking is a risk factor for inducing COPD and most COPD patients are combined with smoking, our focus in this study was biased to whether the patient developed COPD and to search for possible biomarkers of COPD. Next, we will continue to collect samples, focus on whether COPD patients smoke and control subjects smoke, and further study the pathological mechanisms of smoking in the occurrence and development of COPD. Moreover, RNA regulatory networks are only based on bioinformatics predictions, lacking actual experiments to verify, which requires *in vivo* animal experiments and *in vitro* cell models for in-depth investigation. Finally, although several crucial signaling pathways were identified, a series of molecular experiments may help to demonstrate the possible phenotype and pathway regulation of these predictive genes in COPD.

In conclusion, whole-transcriptome sequencing provided all-side data for lncRNA, circRNA, miRNA, and mRNA from COPD samples, discovered lots of differentially expressed RNAs and significant pathways. Based on these ncRNAs, we conducted a series of analyses, which may contribute to discover potential biomarkers in the occurrence and development of COPD, and provide possible therapeutic targets for the diagnosis and prognosis of COPD.

## Data Availability

The data presented in this study can be found in Nutcloud, using the following link: https://www.jianguoyun.com/p/DT8yCdEQi-SpCxiwmPIEIAA.
