# Supplementary material for: Integrative analysis of the expression profiles of whole coding and non-coding RNA transcriptomes and construction of the competing endogenous RNA networks for chronic obstructive pulmonary disease
Source: Front Genet. 2023 Jan 30;14:1050783. doi: 10.3389/fgene.2023.1050783 (PMC9923003; doi:10.3389/fgene.2023.1050783)
Supplement: Supplementary file 1 [file Presentation1.pdf]

Supplementary Material

1 Supplementary Tables

Supplementary Table 1. Primer sequences for qRT-PCR validation of hub gene

Hub Gene	Forward Primer	Reverse Primer	Tm(°C)	Product Length
RPLP0	CGAAGCCACGCTGCTGAACAT	CAGGGTTGTAGATGCTGCCATTGT	60	95
RPL5	GCAGAAGTACATCGGAAGCACATCA	CATCATGTCTGGAGTTACGCTGTTCT	60	135
RPL23	GGTACATCCAGCAGTGGTCATTCG	GCTACTGGTCCTGTAATGGCAGAAC	60	141
RPL35A	TTGAAGGTGTTTACGCCCGAGATG	GCCAGGAGTGACTGTGTTGTTCTT	60	95
RPL13A	GGCTCACGAGGTTGGCTGGA	CGGTAGTGGATCTTGGCTTTCTCTTT	60	84
FAU	CCAGGAAACGGTCGCCCAGA	GCCAGGAGCACGACTTGATCTTC	60	81
RPL27A	AGTGAACAGACACGGGTGAATGC	CCCTTTCCCAGAACTTTGTAGTAGCC	60	98
RPL32	GGAGCTGGAAGTGCTGCTGATG	CGATGGCTTTGCGGTTCTTGGA	60	86
RPS21	GCCAAGGACCACGCATCCATC	TCATCTGACTCACCCATCCTACGAAT	60	125
RPS11	GGAGGCTATTGAGGGCACCTACA	CAGATAGTCTCGGCGGATGACAATG	60	136
GAPDH	CAGGAGGCATTGCTGATGAT	GAAGGCTGGGGCTCATTT	60	138

Supplementary Table 2 GO enrichment analysis of differentially expressed genes

ONTOLOGY	ID	Description	GeneRatio	BgRatio	pvalue	p.adjust	q-value
BP	GO:0006289	nucleotide-excision repair	28/1442	109/18670	9.91e-09	5.76e-05	5.07e-05
BP	c	transcription-coupled nucleotide-excision repair	19/1442	73/18670	1.85e-06	0.005	0.005
BP	GO:0034330	cell junction organization	45/1442	290/18670	5.37e-06	0.009	0.008

ONTOLOGY	ID	Description	GeneRatio	BgRatio	pvalue	p.adjust	q-value
BP	GO:0034329	cell junction assembly	39/1442	241/18670	8.20e-06	0.009	0.008
BP	GO:0032970	regulation of actin filament-based process	55/1442	388/18670	8.47e-06	0.009	0.008
CC	GO:0005925	focal adhesion	53/1489	405/19717	6.16e-05	0.018	0.016
CC	GO:0005924	cell-substrate adherens junction	53/1489	408/19717	7.49e-05	0.018	0.016
CC	GO:0030055	cell-substrate junction	53/1489	412/19717	9.67e-05	0.018	0.016
CC	GO:0005911	cell-cell junction	57/1489	459/19717	1.41e-04	0.018	0.016
CC	GO:1904949	ATPase complex	19/1489	101/19717	1.78e-04	0.018	0.016
MF	GO:0031267	small GTPase binding	64/1443	443/17697	4.93e-06	0.005	0.005
MF	GO:0017016	Ras GTPase binding	61/1443	429/17697	1.35e-05	0.007	0.007
MF	GO:0031625	ubiquitin protein ligase binding	44/1443	290/17697	4.48e-05	0.012	0.011
MF	GO:0044389	ubiquitin-like protein ligase binding	46/1443	308/17697	4.52e-05	0.012	0.011
MF	GO:0045296	cadherin binding	47/1443	331/17697	1.31e-04	0.028	0.026

Supplementary Table 3 KEGG enrichment analysis of differentially expressed mRNAs

ONTOLOGY	ID	Description	GeneRatio	BgRatio	pvalue	p.adjust	qvalue

ONTOLOGY	ID	Description	GeneRatio	BgRatio	pvalue	p.adjust	qvalue
KEGG	hsa03410	Base excision repair	10/691	33/8076	2.96e-04	0.041	0.037
KEGG	hsa05416	Viral myocarditis	14/691	60/8076	4.17e-04	0.041	0.037
KEGG	hsa04216	Ferroptosis	11/691	41/8076	4.82e-04	0.041	0.037
KEGG	hsa05135	Yersinia infection	24/691	137/8076	5.27e-04	0.041	0.037
KEGG	hsa05166	Human T-cell leukemia virus 1 infection	33/691	219/8076	9.09e-04	0.056	0.050

Supplementary Table 4 GSEA enrichment analysis

ID	setSize	enrichmentScore	NES	pvalue	qvalues
HALLMARK_TNF-α_SIGNALING_VIA_NF-κB	189	-0.40842	-1.67988	0.001712	0.034364
HALLMARK_INTERFERON_GAMMA_RESPONSE	187	-0.39784	-1.63049	0.001718	0.034364
HALLMARK_INFLAMMATORY_RESPONSE	177	-0.36267	-1.47303	0.006993	0.082015
HALLMARK_UNFOLDED_PROTEIN_RESPONSE	103	-0.43432	-1.64585	0.009174	0.082015
HALLMARK_MTORC1_SIGNALING	186	0.307609	1.327885	0.011737	0.082015
HALLMARK_ESTROGEN_RESPONSE_LATE	160	-0.35511	-1.42652	0.012302	0.082015
HALLMARK_IL6/JAK/STAT3_SIGNALING	80	-0.40458	-1.46007	0.023301	0.133148
HALLMARK_INTERFERON_ALPHA_RESPONSE	93	-0.37643	-1.39701	0.041746	0.155085
HALLMARK_ANDROGEN_RESPONSE	94	-0.36718	-1.36196	0.043893	0.155085
HALLMARK_P53_PATHWAY	181	-0.31973	-1.29694	0.047872	0.155085
HALLMARK_ADIPOGENESIS	186	-0.31752	-1.29859	0.048611	0.155085
HALLMARK_ANGIOGENESIS	30	0.486584	1.493975	0.049251	0.155085

Supplementary information

The data presented in the study are deposited in the Nutcloud repository, accession link is https://www.jianguoyun.com/p/DT8yCdEQi-SpCxiwmPIEIAA.
